# Detection of Microbial Contaminants in Water: Conventional Methods, Pragmatic Alternatives, and Nanosensing Techniques

**DOI:** 10.1002/mbo3.70057

**Published:** 2025-12-08

**Authors:** Adeyemi O. Adeeyo, Joshua N. Edokpayi, Mercy A. Alabi, Joshua A. Oyetade, Eunice Ubomba‐Jaswa, Penny Jaca, Rachel Makungo

**Affiliations:** ^1^ Department of Earth Sciences, Faculty of Science, Engineering, and Agriculture University of Venda Thohoyandou South Africa; ^2^ Department of Ecology and Resources Management, Faculty of Science, Engineering and Agriculture University of Venda Thohoyandou South Africa; ^3^ Department of Microbiology, School of Life Sciences University of KwaZulu‐Natal Durban South Africa; ^4^ School of Material, Energy, Water and Environment Science Nelson Mandela African Institute of Science and Technology Arusha Tanzania; ^5^ Water Research Commission Water Research Commission Lynnwood Manor South Africa

**Keywords:** contaminants, microbial detection, nanosensors, pathogens, water analysis

## Abstract

The complexities of microbial detection and conventional enumeration necessitates the adoption of pragmatic alternatives. This review expands the boundaries of knowledge for microbial detection and sensing, particularly within the field of water quality analysis. Observed alternatives to conventional techniques for microbial analyses in recent studies include Microarray, Fluorescent in situ hybridization (FISH), loop‐mediated isothermal amplification (LAMP), matrix‐assisted laser desorption ionization‐time of flight (MALDI‐TOF) and flow cytometry, while nanosensors stood out as an alternative for microbial detection in real‐time. This study presents the limitation of conventional methods of detection in water and presents nanoparticles as a detection agent with possibility of incorporation into point‐of‐use detection. It is notable that nanosensors are currently emerging in the detection of bacteria, viruses and other pathogens in water and have been used in the detection of bacterial pathogens than viral. Nanosensors are established as good choice for rapid water analysis with application in point‐of‐use and analytical devices. In the use of nanozymes, the choice over natural enzymes can be linked to their unique and excellent catalytic activities, cost‐effectiveness and ease of mass production.

## Graphical abstract

Conventional culture and immunological methods of detecting bacterial water contaminants have some limitations. There are alternative pragmatic methods which are more efficient and faster in the detection of bacteria in water, especially nanosensors.

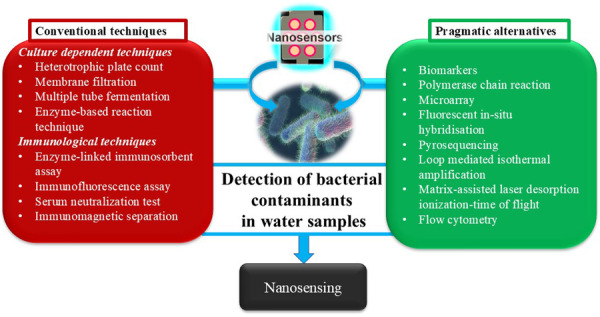

## 1 Introduction

For proper human existence, safe water is essential (Okafor et al. 2024). Unfortunately, billions of people have limited access to safe and clean water which is expected to increase exponentially until the needed effort is geared toward water safety (Salehi 2022). Definite activities and processes that account for significant alteration in water quality are natural processes, agricultural and anthropogenic activities. These activities are mostly reported in developing countries but do not exclude developed nations (Sharma and Bhattacharya 2017). An estimate of 3% of total water available accounts for fresh water while only 0.06% is easily accessible (Musie and Gonfa 2023). Water shortage can impact food production, cause a disturbance in economic growth and may result in negative impact on ecosystem function and services (Berberich et al. 2019). Waterborne diseases have been on the increase due to the problem of water contamination, especially in developing nations (Ali et al. 2025; Kumar et al. 2018). According to the UN/UNESCO report, about 5–10 million deaths are linked to waterborne diseases annually, with children mostly impacted (Ahuja 2015).

The diversities of water pollution problems create difficulty in the adoption of a singular approach to tackle water quality challenges (Abbaspour 2011). Generally, pollution which predominantly involves water had reached a critical point in the earth's ecosystem due to anthropogenic and industrial activities such as human sewage seepage and the leaching of oil or minerals into underground water (Araújo et al. 2022; Gallareta‐Olivares et al. 2023). Among the numerous water contaminants, the recalcitrant category contributing appreciably to water contamination are basically pharmaceuticals and personal care products (PPCs), dyes as well as pesticides (Oyetade et al. 2023, 2022).

Demand for groundwater has increased due to the changes in industrial, agricultural and domestic requirements and have raised concern as awareness of its contamination is usually after substantial concentration of the contaminants have entered the aquifer (Kristanti et al. 2022). Animal manure from agricultural lands is a major source of anthropogenic microbial contamination of groundwater. Other common sources include septic tanks and privies; underground storage tanks, landfills and unauthorized dump sites. Groundwater contamination may also arise from agriculture, unlined drains and illegal waste disposal sites in urban areas (Some et al. 2021). The use of untreated wastewater for irrigation of crops, particularly those consumed raw, can also result in exposure to pathogens and infection (Onyango et al. 2018). Many of these microbes contaminating water remain infective for long periods in water and can be distributed over long distance from their source (Chow 2010). As waterborne pathogens travel through the environment they are typically diluted to low, but clinically concerning concentrations creating a detection challenge (Danielopol et al. 2003). All these raise concerns and the necessity for effective means of detection of microbial contaminants in water.

Water contamination from microbial origin, usually from fecal sources, has been identified to have deleterious effects (Hamilton et al. 2006; Kumar et al. 2014). These microorganisms with adverse health impacts includes pathogenic bacteria, viruses, fungi, algae, protozoa and parasites (Sharma and Bhattacharya 2017). During erosion and run off of rainwater, they get into various water banks such as lakes, streams, oceans and underground water, which are the main supply to the communities (Fong and Lipp 2005). They cause diseases such as diarrhea, anemia, cholera, typhoid, gastroenteritis, salmonellosis, shigellosis, giardiasis and cyclosporiasis. Examples of such bacteria are *Vibrio cholerae, Escherichia coli, Salmonella typhi* and viruses are adenovirus, astrovirus, hepatitis A and E viruses, rotavirus, norovirus, caliciviruses and enteroviruses (Kumar et al. 2014). Children are the most affected in the occurrence of water contamination with estimated 1.7 billion children and approximate mortality rate of 31% (Hamilton et al. 2006; Kumar et al. 2014). Major contaminants of water bodies are Norovirus, *Campylobacter, Cryptosporidium, Giardia* and *E. coli* (Borchardt et al. 2007) and microbial indicators that are more resistant than *E. coli* such as intestinal Enterococci or *Clostridium perfringes* can be used to supplement water quality assessment (Connelly and Baeumner 2012).

Although more reported in developing countries, waterborne pathogens are also a problem in developed nations (Howe et al. 2002). The annual number of cases of acute gastrointestinal illness in the United States caused by the consumption of contaminated water has been estimated to be from 4.26 million to as high as 32.9 million (Figueras and Borrego 2010; Pitkänen et al. 2011) with more than $1.23 billion spent by the US government since 2004 on the cleanup of toxic pollutants in waterways around the Great Lakes region alone (Duke University 2023). Waterborne pathogens which persist in the environment for a long duration eventually get diluted to low numbers and thus difficult to detect. It is therefore pertinent that waterborne microbes be detected if water safety will be adequately monitored. Lack of access to potable water when combined with poor hygiene and lack of sanitation facilities can cause great health challenges (Schijven et al. 2010).

These global challenges necessitate proactive detection techniques involving quality assessment and monitoring, and quantitative surveillance of microbial risk for prompt mitigations and remedial actions. Although, there are traditional methods of detection involving culturing on appropriate agar; however, the time required, sensitivity and waste generation are of concern (Hrudey and Hrudey 2007). More advanced methods such as microscopy and polymerase chain reaction (PCR) have cost and complexity disadvantages (Howe et al. 2002). Other molecular techniques such as ATP as biomarker, microarray, fluorescent in‐situ hybridization (FISH), pyrosequencing and flow cytometry have been employed for the detection of contaminants of microbial origin. Although sensitive and specific, these methods are also costly (Howe et al. 2002). Also, the detection of microbial presence in water is challenging owing to physical disparity in the major pathogen groups and the low concentration of these waterborne pathogens in large volume of water (Colford et al. 2006; Deshmukh et al. 2016).

Recently, nanotechnology has found application in water treatment and nanozymes are being explored in next generation biosensing. Nanozymes function like enzymes and are cheap, easily produced, highly stable and small‐sized. They also possess distinct biological, chemical, mechanical and physical properties (Messner et al. 2006). With the climaxing application of nanozymes, nanosensors offer the potential for the real‐time detection of microbial contamination in water (Howe et al. 2002). This review will contribute to the expansion of the frontiers of microbial detection and alternatives for microbial enumeration and signal amplification of microbial pollutants have been explored via intensive literature study.

## 2 Methodology

Articles used in this study were obtained from research websites such as Google Scholar, PubMed, Science Direct, Scopus and Web of Science. Articles with information on water quality monitoring, methods of detecting microbial contaminants, biosensors, nanosensors and point‐of‐use detection of contaminants were obtained and reviewed. A total of 242 articles were downloaded for the purpose of this review, 97 articles were excluded because they do not address the general use of sensors, biosensors, nanozymes and/or nanosensors in the detection of water contaminants, and a total of 161 articles are used in this review paper spanning from 2001 to 2025.

## 3 Conventional Techniques for Microbial Detection in Water Samples and Systems

**Figure 1:**
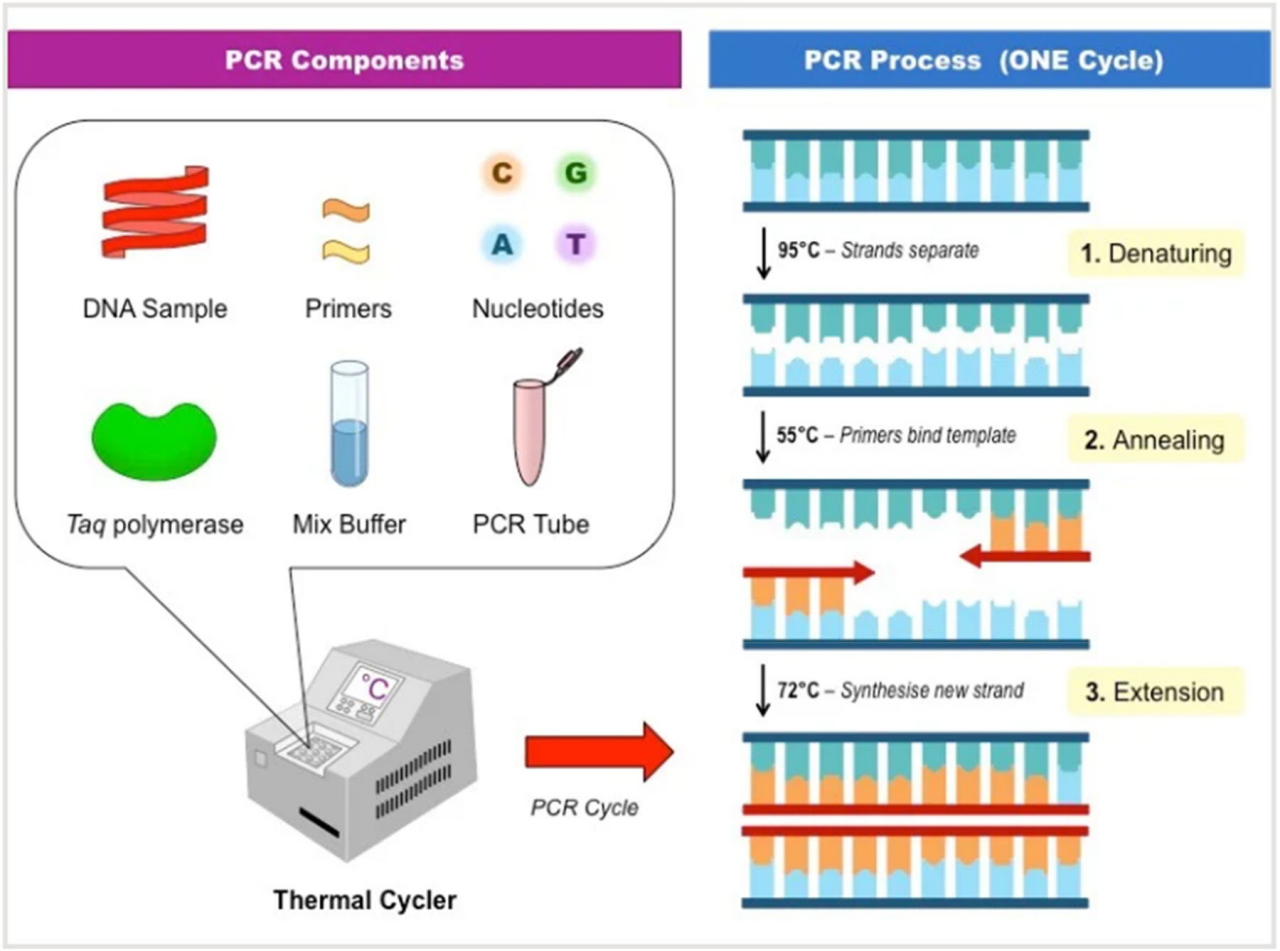
The process for polymerase chain reaction (Takahashi 2018).

Effective water analysis techniques are needed to lessen the health risks posed by consumption of contaminated water (Pandey et al. 2014). To detect contaminants, cultural and immunological molecular techniques have been employed (Table 1). The culture‐dependent approaches are based on the development, isolation, and identification of pathogens on certain media (Figure 1) (Miagostovich et al. 2008; Yu et al. 2017). This culture‐dependent techniques are used in the detection of microbial water contaminants such as coliform bacteria (*Escherichia, Citrobacter*, and *Enterobacter*) which are habitual inhabitant of feces and can be used to infer fecal contamination of water (Jung et al. 2014; Shanker et al. 2020; Tambi et al. 2023). Heterotrophic plate counting, membrane filtering, and multiple tube fermentation are a few examples of culture‐dependent methods (Howe et al. 2002; Staley et al. 2012). Indicators for fecal contamination include *Clostridium perfringes*, which can also be a sign of other persistent organisms such as viruses and protozoa (Liang and Yan 2019). Enzyme‐based reactions are also used in the detection of microorganism, allowing the change in color of the enzyme in the presence of the organism of interest.

**Table 1:** Methods of microbial detection, their advantages and disadvantages (Ekelozie and Obeagu 2018; Howe et al. 2002; Ramírez‐Castillo et al. 2015; Velusamy et al. 2010).

Method	Types	Advantages	Disadvantages
Culture dependent	Heterotrophic plate count	Simple, relatively low cost, allows both quantitative and qualitative information, safe to execute	Laborious and time consuming, false positive results are frequently obtained, cannot detect non‐culturable cells
	Membrane filtration	Same as in heterotrophic plate count	Same as in heterotrophic plate count
	Multiple tube fermentation	Same as in heterotrophic plate count	Same as in heterotrophic plate count
	Enzyme‐based reaction technique	It can detect non‐cultivable coliforms	More expensive
Immunological	Enzyme‐linked Immunosorbent Assay, Immunofluorescence Assay, Serum Neutralization Tests, Immunomagnetic Separation	Specific, can detect bacteria toxin, can run multiple samples at a time, less time consuming compared to culture‐based assay. ELISA has availability of extensive database	Cross reactivity, possibility of false negative result, need for pre‐enrichment, limited by the low concentration of several water pathogens

Microorganism detection has also utilized some immunology‐based techniques which involves the binding of antigens and antibodies (Altintas et al. 2015; Ferrer et al. 2015). Antigens are proteins or polysaccharides which are capable of stimulating immune response. Antibodies can be monoclonal or polyclonal and combine specifically with antigens (Yang et al. 2019). There is specificity in the attachment of antibodies to antigen and this is the mechanism with which immunological assays operate. Methods based on immunology assess the number of bacteria in a solution using the idea that antibodies attach to antigens specifically. An advantage of immunological methods over cultural method is in its specificity and reduced time of analysis (Boubetra et al. 2011). Different immunological detection methods include serum neutralization tests, enzyme‐linked immunosorbent assay, immunomagnetic separation and immunofluorescence assay (Douterelo et al. 2014; Howe et al. 2002). However, immunological methods present disadvantages including low sensitivity, false positive results, cross‐reactivity with closely related antigens, pre‐enrichment to produce cell surface antigen and knowledgeable personnel (Yang et al. 2019).

## 4 Pragmatic Alternatives for Microbial Detection in Water

### 4.1 Biomarkers

The presence of microorganism in a sample can be detected using biomarkers such as nucleic acid, proteins, adenosine triphosphate (ATP), antigens and metabolic products (Law et al. 2015). In addition to holding the genetic material and producing proteins in a cell, nucleic acid also contains nucleotides. Individual bacteria have unique nucleic acid sequences that can reveal the type of microorganism present (Nurliyana et al. 2018). Microorganisms express proteins on their surfaces, and these proteins are frequently utilized as biomarkers in medical diagnoses. They function for the movement of metabolites between cells and the exchange of chemical signals (Hsu et al. 2014).

The proteins expressed by these microorganisms are worked on by aptamers which act as ligand for the extraction and purification of targeted surface protein, these antibodies are able to recognize the membrane protein on the cell surface in a natural environment (Azmi et al. 2017). Pathogens have antigens which specifically bind to antibodies, hence, antibodies can be used to detect pathogens and diagnose infections (Omar and Barnard 2014). Additionally, microorganism's metabolic activity results in the production of metabolites such as amino acids and organic acids, which can then be released depending on the metabolic status of the microbes in the sample and thus used in detecting the presence or estimating the activity of microorganism (Jayan et al. 2020).

Also, the coenzyme ATP which is a living cell's biological fuel can be utilized to forecast cell viability and damage. It plays a significant function in cellular metabolism. The amount of ATP produced indicates how many cells are metabolically active, and this information can be used to estimate the number of live cells (Yu et al. 2019). The development of a microfluidic system which can act as an online early warning system for measuring microbial contamination in drinking water samples through quantification of ATP has been tested. The presence of bacterial contamination in lake, rain and spiked tap water was analyzed using ATP as a biomarker and *E. coli* suspension as reference (Hansen et al. 2019).

### 4.2 Polymerase Chain Reaction (PCR)

The concept of hybridization and amplification is used in nucleic acid‐based methods of detection. The target analyte can interact with a particular nucleic acid acting as the probe, allowing for its detection. Due to its great sensitivity and speed, PCR is mostly chosen for the identification of microorganisms. PCR is one of the most popular molecular‐based techniques for detecting aquatic microorganisms (Yang et al. 2019). It relies on the in vitro amplification of DNA and result is obtained rapidly (Figure 1). The traditional PCR, however, is unable to distinguish between live and dead cells and it is a difficult process (Palecek 2002). There are different types of PCR including droplet digital PCR, multiplex polymerase PCR and quantitative PCR (Table 2) (Howe et al. 2002).

**Table 2:** Types of polymerase chain reaction, advantages and disadvantages (Hyun et al. 2018; Lakhin et al. 2013; Slavov et al. 2017).

Methods	Advantages	Disadvantages
Conventional PCR	Highly specific, rapid, can be automated	Does not differentiate between viable and nonviable cells, sensitive to PCR inhibitors, complex process
Reverse Transcriptase PCR	Can differentiate between viable and nonviable cells, highly specific, rapid, possibility of multiplexing, can detect multiple pathogens	Sensitive to PCR inhibitors, possibility of cross contamination, primer design is very important
Multiplex PCR	Highly specific, can be automated, multiple pathogens can be detected	Primer design is crucial, vulnerable to PCR inhibitors, unable to distinguish between viable and nonviable cells
Quantitative PCR	Enables quantification of DNA targets, highly specific, rapid, minimizes risk of cross contamination, no need for post‐PCR analysis	Can detect only one pathogen at a time, cannot detect damaged genomes
Droplet Digital PCR	Highly specific, direct enumeration of labeled cells	Less accurate quantification of larger amplicons, low specimen throughput, more expensive, higher risk of contamination

Although conventional PCR has the limitation of differentiating between viable and dead cells, advanced PCR protocols cover this limitation. The two reaction phases of the viability polymerase chain reaction (vPCR) technology—PCR amplification and nucleic acid intercalating dye pretreatment—allow for the rapid separation of viable bacteria from dead ones in a sample (Chen et al. 2022). Light‐activated nucleic acids embedded in dyes, such as ethidium monoazide (EMA) or propidium monoazide (PMA), can enter cells and form an irreversible binding with DNA molecules because of the incomplete cell membrane of damaged or dead bacteria. This prevents further PCR amplification to distinguish between living and dead bacteria (Rayo et al. 2025). This also helps in the identification of the presence of viable but non‐culturable bacteria (Reichelt et al. 2023).

PCR was used for the detection of *Listeria monocytogenes*, *E. coli* O157:H7 and *Salmonella* species in urban water samples from different sampling points in Chambo Canton, Ecuador. Among the 50 points studied, *E. coli* was detected in 40 samples (80%), *L. monocytogenes* in 20 (40%), and *Salmonella* spp. in 10 samples (10%). The study, therefore, suggested that the studied water samples may potentially transmit diseases such as typhoid fever, paratyphoid fever, and hepatitis A (Darwin Núñez 2025). Rapid quantitative PCR was used for the detection of *S. dysenteriae*, *V. cholerae*, *S. typhimurium* and *E. coli* in different surface water samples (Gao et al. 2024).

The use of qPCR as a routine method of contaminant detection is often credited to its speed, sensitivity and capacity to measure microbial populations. Contamination is detected in a few hours, and this makes it a useful technique for determining the possible health hazards linked to bacterial contamination and evaluating quality of water (Saleem et al. 2023). It has been used for monitoring wastewater treatment plants and water facilities for the presence of *E. coli, Legionella*, norovirus and adenovirus and is useful for the early detection of outbreaks (Oon et al. 2023). It can detect low levels of bacteria, viruses and protozoa. The US EPA method 1609.1, which is a type of qPCR monitoring, is a rapid method for the routine detection of fecal indicators in recreational water (Saleem et al. 2023).

### 4.3 Microarray

This is a multiplex lab‐on‐a‐chip that evaluates massive volumes of biological material using high‐throughput screening techniques. It could be a protein, an antibody or a DNA microarray. DNA microarrays are potent genomic technologies that are frequently used to characterize microorganisms in environmental samples, detect specific mutations in DNA sequences, and monitor gene expression under various cell growth conditions (Aw and Rose 2012). Nucleic acid hybridization makes it possible to detect hundreds of genes using a test (Tut et al. 2021). Proteins that have been labeled with a fluorescent dye are frequently put to protein microarrays. Protein microarrays require little samples or reagents and are quick, automated, inexpensive and sensitive. Protein microarrays are used to detect antigens by setting a collection of capture antibodies on a solid surface.

The benefit of utilizing a microarray for microbial identification is that it can quickly identify numerous genes at once since it can screen a huge number of sequences and can be automated. Microarrays can be used to assess the resistance of pathogens to antimicrobial drugs. Its limitations, however, include its high cost and inability to distinguish between viable and nonviable cells. Due to nonspecific hybridization, it has low specificity and sensitivity. Higher sensitivity is however obtained when PCR and microarray are coupled, although a considerable amount of sample is required (Altintas et al. 2015). Waterborne pathogens such as E*. coli, S. enterica* Typhimurium (Hajia and Department of Molecular Biology, Health Reference Laboratory, Ministry of Health and Medical Education, Tehran, Iran 2018), *Entamoeba* spp., *Cryptosporidium* sp, *Acanthamoeba* spp. and *Giardia intestinalis* (Kadri 2020) have been detected using microarray.

### 4.4 Fluorescent In Situ Hybridization (FISH)

**Figure 2:**
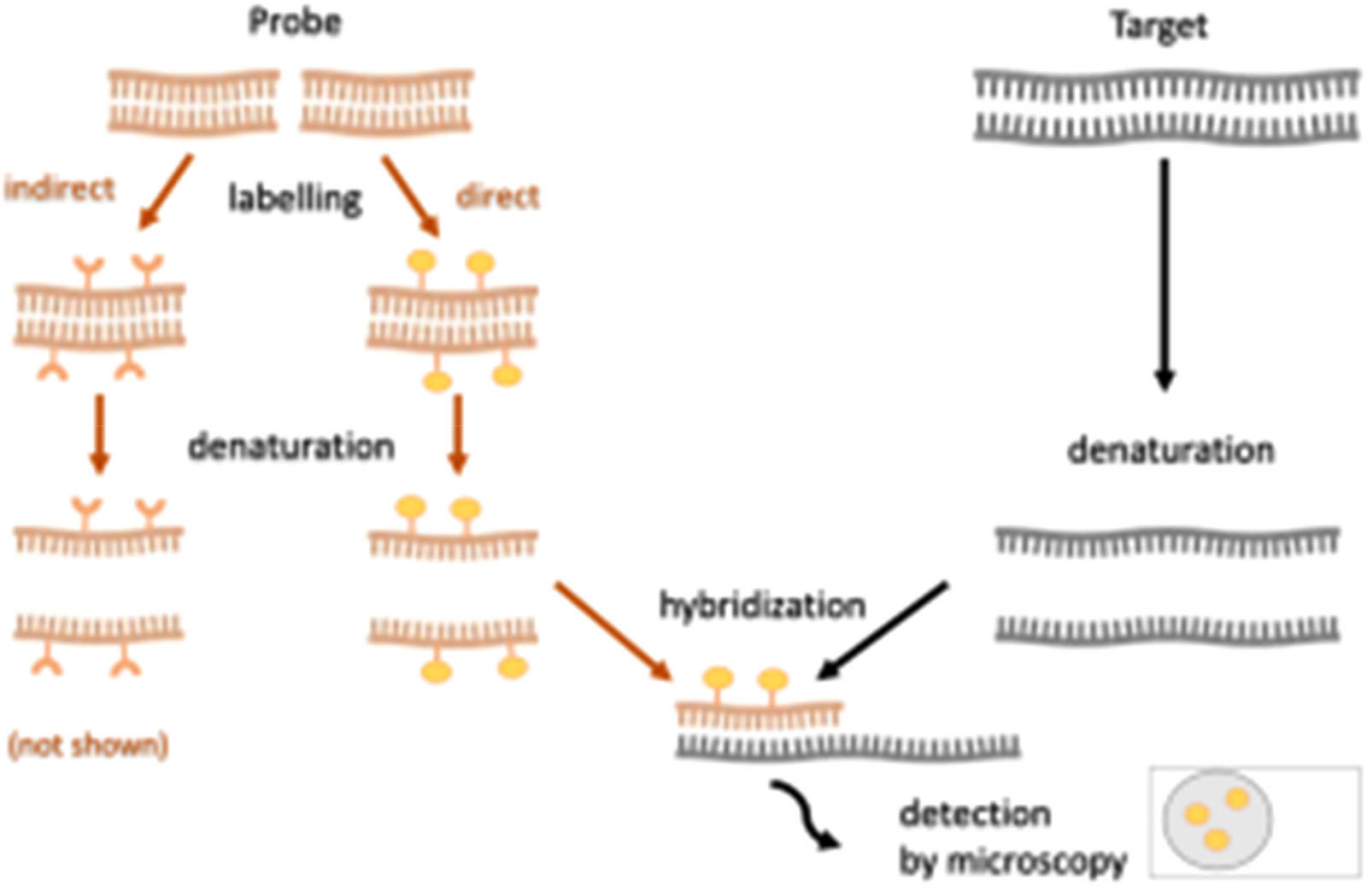
The working principle for fluorescent in situ hybridization (Kushkevych et al. 2021).

FISH involves fixing materials to a slide, followed by permeabilization and incubation (hybridization) with the fluorescently tagged probe (Olusanya et al. 2022). To perform the analysis, a sample is hybridized with a rRNA oligonucleotide probe that has a fluorescent dye attached to one end (Figure 2). FISH helps to identify and gauge the prevalence of a specific microbe in a community and when used with a direct viable count assay, it is able to distinguish between viable and nonviable cells, which is an advantage it has over microarray (Zhou 2003). FISH is effective in the detection of a particular microorganism in a biofilm (Altintas et al. 2015). However, it is limited in that it requires pre‐enrichment and concentration stage which may lead to false negative results. It is also low in sensitivity (Trevino et al. 2007). FISH was used for the detection of *E. coli, K. pneumoniae, E. aerogenes* and *C. freundii* in simulated water and domestic wastewater samples. The optimized FISH method enabled the detection of coliforms within 4 h, a significant improvement over traditional microbiological methods, which required 1 to 2 days for detection. In domestic wastewater, it detected the presence of *E. coli and E. aerogenes* which were not detected by coliform detection kit, multiple tube fermentation and membrane filter (Kuo et al. 2020).

### 4.5 Pyrosequencing

**Figure 3:**
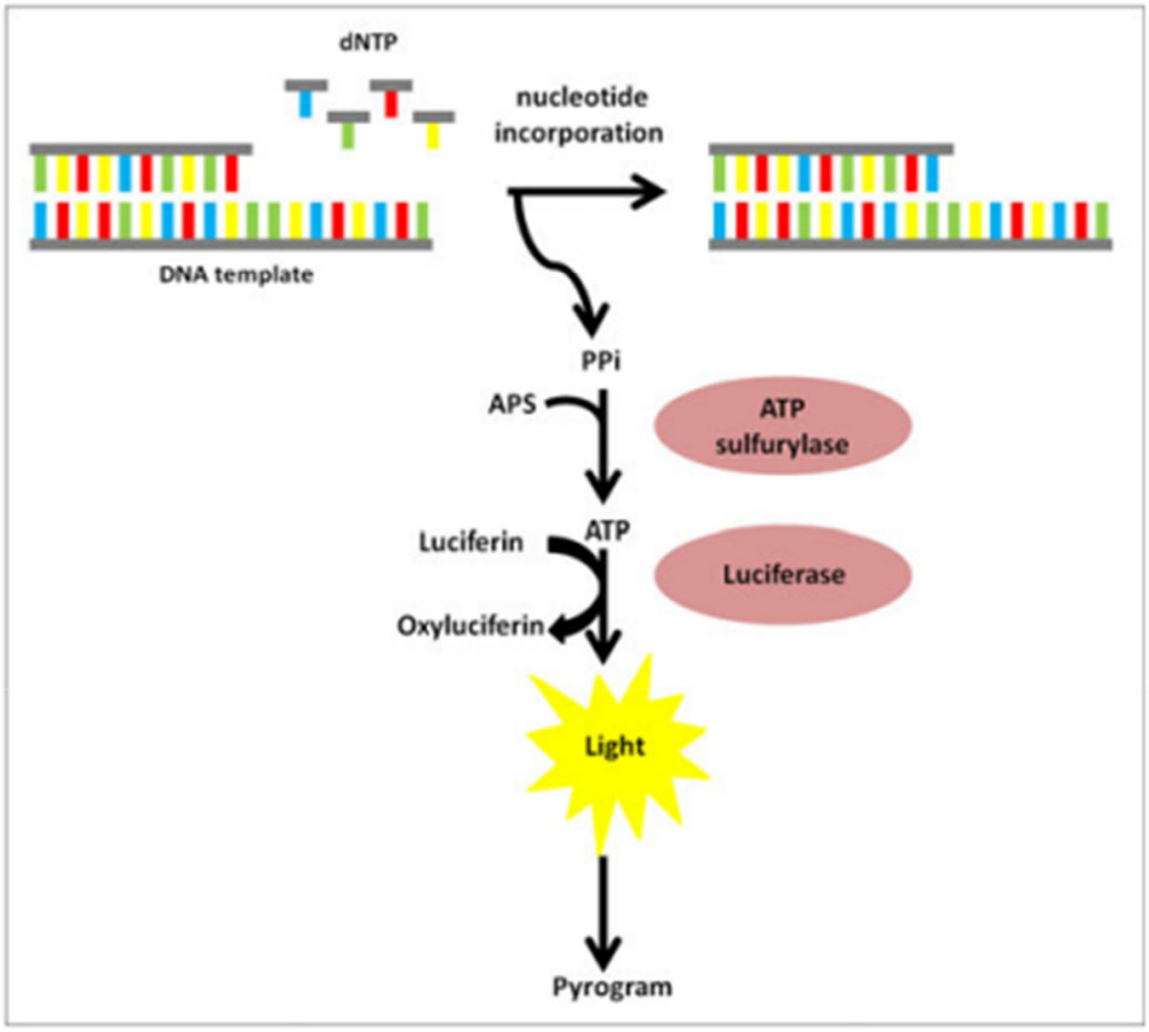
The process of detection using pyrosequencing (Rybicka et al. 2016).

Pyrosequencing is a technique for short‐read DNA sequencing that employs bioluminescence and an enzyme‐couple reaction to continuously track the pyrophosphate release that goes along with nucleotide incorporation (Ahmadian et al. 2006). The enzymes DNA polymerase, ATP sulfurylase, luciferase, and apyrase are needed for pyrosequencing (Figure 3), which reads a high number of sequences in a single run (Siqueira et al. 2012). An advantage of pyrosequencing is that it can identify novel pathogens and detect multiple aetiologies. However, it also requires high concentration of DNA making its sensitivity limited. Pyrosequencing is also costly, complex and data generation may be less efficient (Gong et al. 2010). Janzen et al. (2015) used pyrosequencing in the detection of *Bacillus anthracis* in bottled water. In the study, based on the read lengths acquired from pyrosequencing, the chromosomal and plasmid targets that were identified as specific for *B. anthracis* were contrasted. Across all 20 strains of the bacterial strains tested, the average length for each target was 48 bp for acpB, 68 bp for gerXB, 42 bp for capBCAD, 64 bp for cya, 36 bp for prophage lambda1 and 54 bp for prophage lambda3.

### 4.6 Loop Mediated Isothermal Amplification (LAMP)

This is the most used isothermal amplification technique. The LAMP reaction achieves high sequence selectivity by employing strand‐displacement DNA polymerase and a series of up to three primer pairs that bind to six different sites on the target DNA (Girish and Karabasanavar 2020). LAMP is a highly specific detection method that can be completed at about 65°C owing to the amplification mechanism, which replaces the first denaturation phase and requires no temperature changes during the reaction (Nagamine et al. 2001). A DNA‐intercalating fluorescent dye can quickly make the LAMP products visible to the naked eye since LAMP creates far more product DNA than PCR does (Martzy et al. 2017). A number of LAMP tests for the identification of waterborne infections have been developed such as *Naegleria fowleri* (Mahittikorn et al. 2015), Acanthamoeba (Mahmoudi et al. 2015) and *Enterococcus faecalis* (Ahmad et al. 2017; Wang et al. 2017). Water samples were collected from a beach, a freshwater reservoir, and from two sewer manholes in Singapore and assessed for contamination by fecal indicator bacteria using LAMP. Results showed the presence of *E. coli* and *Enterococcus* species (Fu et al. 2021).

### 4.7 Matrix‐Assisted Laser Desorption Ionization‐Time of Flight (MALDI‐TOF)

**Figure 4:**
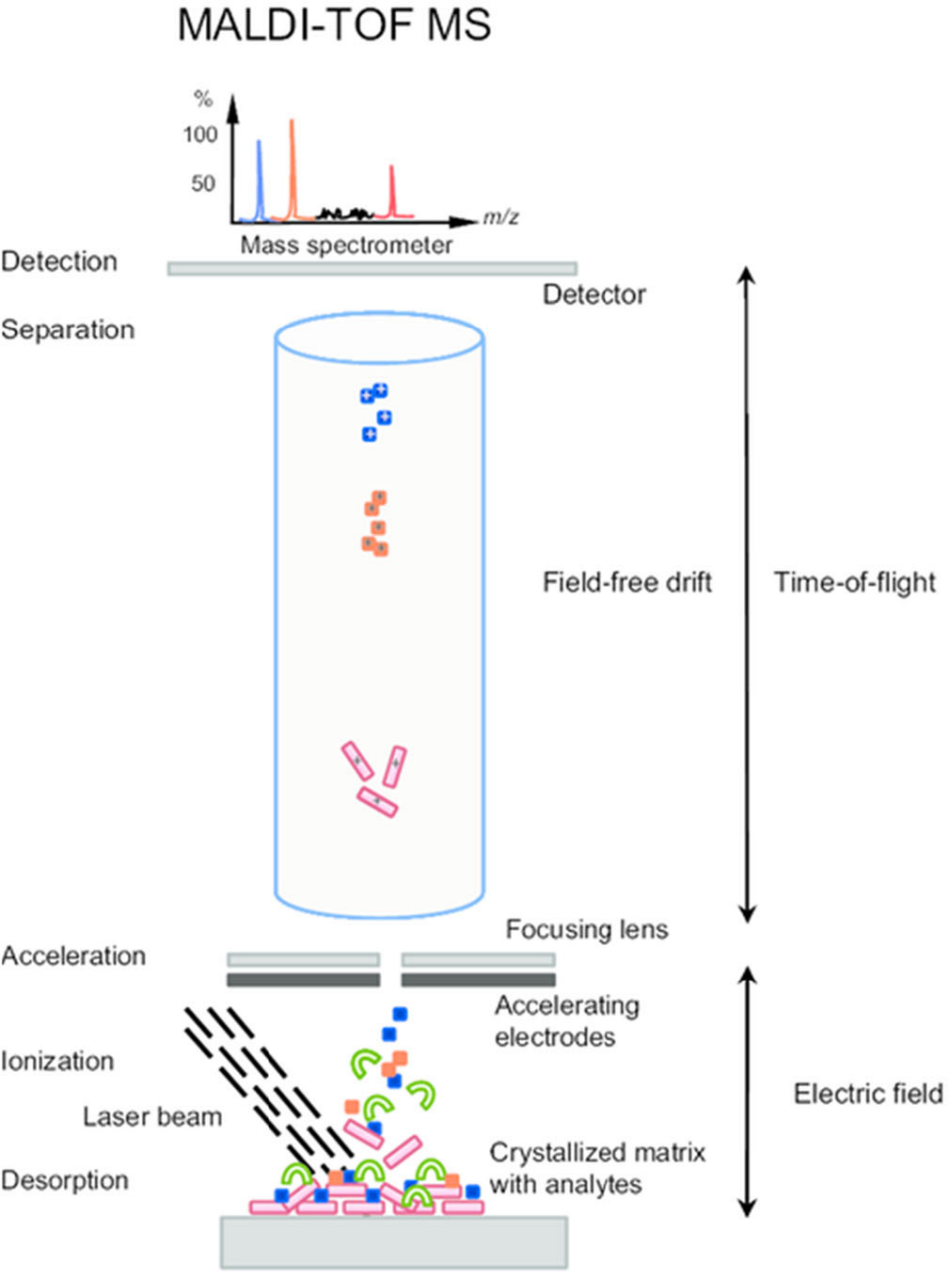
The process of detection using MALDI‐TOF (Lavigne et al. 2013).

The MALDI‐TOF is a high‐throughput technology that uses a range of algorithms built into commercially available instruments to match the protein fingerprints produced by microbial cells to a library of reference spectra (Calderaro et al. 2014). It enables the use of ions produced during the analysis to find biocomponents (Canciu et al. 2021) (Figure 4). The MALDI‐TOF MS Biotyper system was used to identify *Legionella* spp. in drinking water in Germany, and the findings are encouraging considering that this detection approach is being used as an alternative identification method to evaluate water quality (Dilger et al. 2016). These technologies offer details on the identification and detection of microorganisms in a range of surface water and wastewater samples, and they provide quick and precise results for analysis (Canciu et al. 2021). In the proposed method, there was correct identification of *Legionella* species which corresponded to the result from control detection (Pascale et al. 2020). MALDI‐TOF MS thus demonstrated promise for the quick and precise detection of coliforms in different water samples which agreed so well with 16S rRNA sequencing (Suzuki et al. 2018). While MALDI‐TOF is used in the analysis of water samples, the method relies largely on the need for preculturing of bacteria on growth medium. In clinical and particularly in environmental samples, bacteria rarely exist as single cultures; rather, they mostly occur in mixtures and biofilms, and this presents the challenge of accurate identification. Hence, obtaining pure cultures is very important to the process. Pure cultures are usually grown as pure cultures on solid medium in preparation for the analysis (Topić Popović et al. 2023). *P. stutzeri* and *Acinetobacter haemolyticus* were isolated from well samples collected from private and public water supply wells in Northern Texas (Santos et al. 2017).

### 4.8 Flow Cytometry

An optical detection‐based technique for analyzing individual cells in intricate matrixes is flow cytometry. A sample is injected into a liquid that flows across the instrument's flow detecting area in the process, which can be regarded of as a sort of automated fluorescence microscopy (Han et al. 2016). Microorganisms are suspended in a liquid matrix for microbe identification, which is then exposed to a laser‐focused light beam. This leads to the scattering and absorption of focused incident ray from the microorganism to be detected. The quantity, size, and shape of microorganisms can be estimated based on the scope and characteristics of this scattering process (Ambriz‐Aviña et al. 2014). This approach is advantageous since it is quick and highly sensitive. Flow cytometry is an appreciable technique for the detection of small quantities of certain organisms in fluids, rinses, or full liquid aliquots due to the method's great sensitivity. The various dilution stages needed before analysis in flow cytometry lowers the amount of trash, which will facilitate antibodies' ability to bind to their targets and analyze each individual cell as it passes the site of detection. Unfortunately, this reduces the flow cytometer's potential sensitivity. Additionally, the flow cytometer can provide vast volumes of data, making the additional analyses difficult (Lugli et al. 2010).

In flow cytometry, a combination of dye helps to distinguish between live and dead cells. Propidium iodide is one of the most commonly used in the study of bacterial viability as it stains dead cells (Servain‐Viel et al. 2024) while Calcein violet AM stains live cells (Grandy et al. 2022). These live cells ‐ dead cells dye combination gives better effectiveness to the distinction between live and dead cells (Grandy et al. 2022). Flow cytometry was used for the detection of *Tetrasphaera elongata* and *Pseudomonas* species in water samples collected in the water column of Lake Pavin, France (Bouquet et al. 2025). Additionally, flow cytometry can also be used in the identification of viable but non‐culturable bacteria (Morishige et al. 2015). Flow cytometry has been incorporated as routine method for the detection of bacterial contaminants in water in European countries such as Switzerland. For example, the Swiss companies Metanor (Online Bacteria Analyzer) and Sigrist (BactoSense) succeeded in integrating all the necessary measurement steps into a mobile and autonomous device. This is an add‐on device enabling benchtop flow cytometry to continuously perform measurements is provided by OnCyt (Switzerland) (Schönher et al. 2021).

Owing to the disadvantages of cultural and conventional immunological methods, these alternative methods for the detection of microbes have been used presenting advantages such as specificity and sensitivity, but they have not been without some disadvantages as well (Table 3).

**Table 3:** Advantages and disadvantages of some microbial detection methods.

Detection method	Advantages	Disadvantages
Microarray	Highly specific and sensitive, can detect multiple pathogens	Highly costly, cannot differentiate between viable and nonviable cells, possibility of nonspecific hybridization
Fluorescence In Situ Hybridization	Highly sensitive, can differentiate between viable and nonviable cells	Low sensitivity, requires pre‐enrichment and concentration, possibility of false positive results, costly
Pyrosequencing	Provides large number of sequences in a single run, can detect multiple cells	Needs pre‐concentration of sample, low sensitivity, complex and costly
Loop‐Mediated Isothermal Amplification	Its specificity is extremely high because it can amplify a specific gene by discriminating a single nucleotide difference. The amplification efficiency is very high because there is no time loss of thermal change. LAMP assays are significantly rapid, and do not require expensive reagents or instruments	The method is complex. It requires a complex primer design system which can constrain target site selection and resolution or specificity
Matrix‐assisted Laser Desorption Ionization‐time of Flight	Speed of analysis, low sample volume, high sensitivity, ease of use, inexpensive consumables, and wide mass range coverage	The inability to discriminate between related species which can be due to the inherent similarity of the organisms themselves
Flow cytometry	It allows for the analysis of multiple parameters across a large number of cells	They cannot analyze cells that are not suspended in culture The laser can only analyze one cell at a time. Requires technical know‐how

## 5 Nanosensing Techniques for the Detection of Microbes in Water Samples and Systems

**Figure 5:**
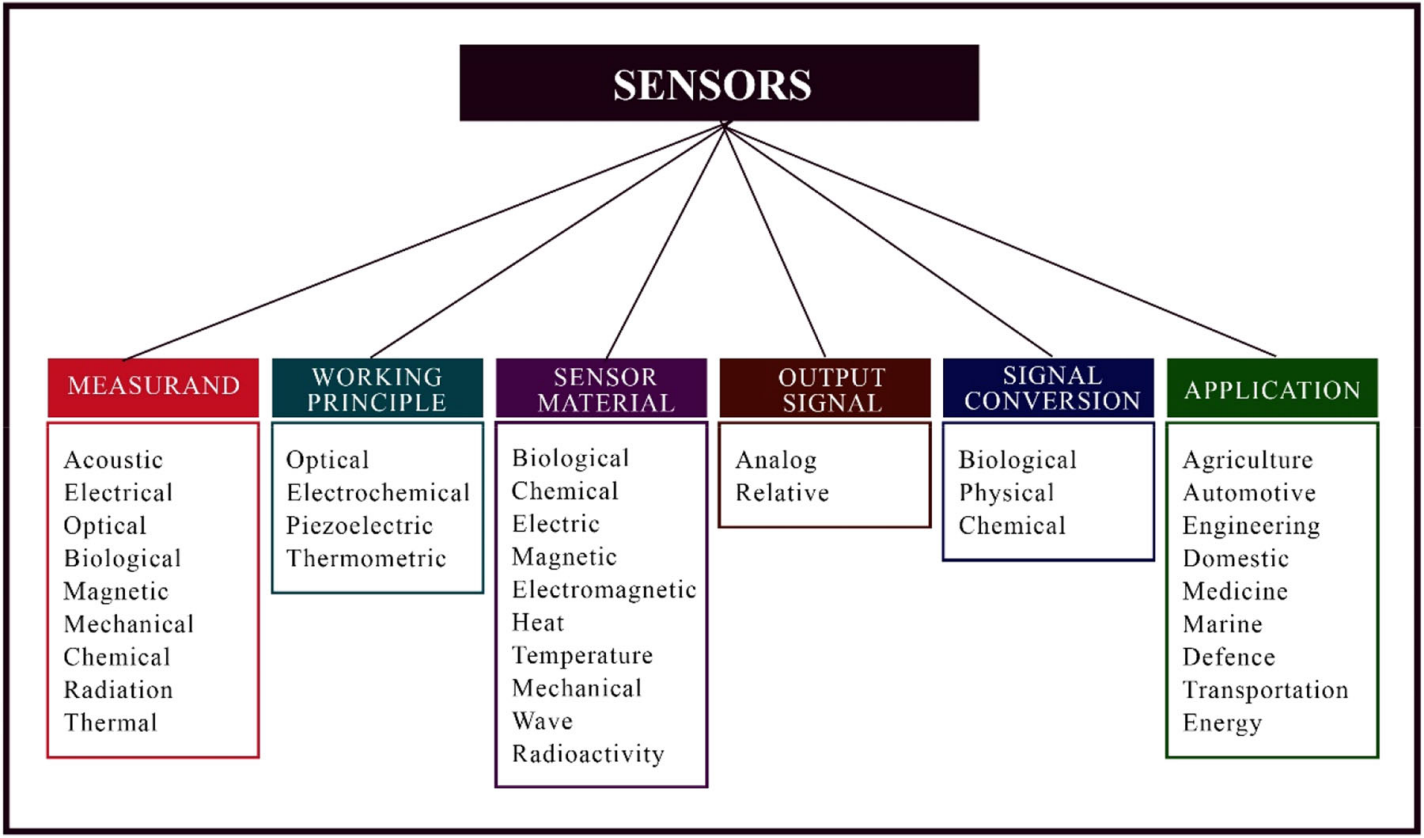
Classification of Sensors.

Sensors have been used in sensing different substances including toxic ions, pesticides, phenolic pollutants, antibiotics residue and cancer cells (Dutta et al. 2012; Guivar et al. 2015; John et al. 2022; Patel et al. 2020; Srivastava et al. 2018). Sensors can also be classified based on the working principle, energy/power, signal conversion, sensor material, output signal, specifications, and applications (Figure 5). The use of nanoparticles in optical, electrochemical, piezoelectric and magnetic biosensors in signal amplification as well as biosensing system, labeling, isolation and quantification of target analyte has been reported by Shinde et al. (Shinde et al. 2012). The increasing use of nanoparticles was also reported by Wu et al. (2021). Nanosensors have been used in the detection of microorganisms using different detection methods (Table S1) and some reports present the use of nanoparticles in the detection of pathogens in food and water. The use of nanosensors in water monitoring as a point‐of‐use for quality monitoring in water and liquid matrices have been demonstrated (Table 4).

**Table 4:** Microbial detection in food and water using nanoparticles.

Nanoparticle	Microbe(s) detected	Transducer type	Sample matrix	Detection limit	References
Bovine serum albumin‐templated Co_3_O_4_ magnetic nanozyme	*S. aureus*	Colourimetric	Milk	8 cfu/mL	Shangguan et al. (2015)
Gold nanoparticle	*E. coli*	Colourimetric and electrochemical	fruit juice	~10 cfu/mL	Das et al. (2020)
Mesoporous core‐shell palladium@platinum nanoparticles	*S. enteritidis, E. coli*	—	milk	~20 cfu/mL–34 cfu/mL	Cheng et al. (2017)
Prussian blue nanoparticle	*S. typhimurium*	Colourimetric	Powdered milk	6 × 103 cfu/mL–6 × 106 cfu/mL	Farka et al. (2018)
Graphene quantum dots	*Y. enterocolitica*	Electrochemical	Milk	5 cfu/mL	Savas and Altintas (2019)
PAMAM‐OH dendrimer	*P. aeruginosa*	Optical	water	10^4^ cfu/mL	Ji et al. (2004)

Also, with the rise in the re‐use of treated wastewater as potable water, there is need for the proper screening of recycled water before use (Vikesland 2018). For instance, research has revealed elevated levels of *Giardia, Cryptosporidium*, and *Legionella* in water distribution systems (Ryu et al. 2007). The successful use of nanomaterial‐enabled sensors in medical and industrial application is an indication that it can be maximized in water monitoring. Water quality monitoring is possible with the use of nanomaterials in sensors as they are of low cost yet will produce rapid result of analysis. Shawon et al. (2020) reports that in comparison to traditional sensors, nanosensors have a number of benefits, including low cost, high sensitivity and selectivity, real‐time detection, high efficiency, portability, and other crucial advantages.

Although there has been a focus on bacterial contamination in water, other pathogens such as protozoa need to be greatly considered in water safety as well as parasitic protozoa such as *Toxoplasma gondii*, *Cyclospora cayetanensis, Blastocystis hominis, Balantidium coli, Acanthamoeba spp., Entamoeba histolytica, Isospora belli, Sarcacyctis spp. and Naegleria spp*. Have been reported to cause illnesses in humans and are spread through water (Omarova et al. 2018).

## 6 Technological Developments and Applications of Nanosensors

Over the past decade, some developments have been recorded with respect to nanosensors. Exploring the ability of metallic nanoparticles as enzyme mimics has limited the use of costly biological elements. They are also better as they are more stable and not easily denatured like natural enzymes. The plasmon properties of nanoparticles have also helped in their activity for point‐of‐use testing (Mohamad et al. 2019).

There have been reasons for collaboration of scientists from different disciplines in the development of biosensors. Also, the possibility of building a database for finding the relationship between nanozyme structure and catalytic performance has helped with the synthesis of nanozymes with the desired function (Wang et al. 2019). Nanozymes have also been linked to computational research for the better understanding of its mechanism of catalytic actions. Discrete Fourier Transform (DFT) investigation has been used in predicting the geometry and energy of the nanomaterials (Wang et al. 2019). Reports have shown molecules which can efficiently boost the catalytic activity of nanozymes (Shah and Singh 2018; Simos et al. 2012).

Point‐of‐use nanosensors have appeared to make it easier to identify various analytes locally. Due to their accessibility, quick turnaround times for analyses, and cost effectiveness, they are replacing culturally‐based and other laborious procedures (Kumar et al. 2019). The detection is dependent on fluorescence, colorimetry and electrochemistry to achieve rapid and easy interpretation of signal. Paper‐based, lateral flow and microfluidic devices are examples of point‐of‐use devices (Kumar et al. 2019). Paper‐based devices are less costly, simple to operate and portable. A paper‐based hand‐held culture device was used in the detection of *E. coli* in water and fluorescence was detected using a portable luminescence imaging devices and the sensor had a limit of detection of less than 10 cfu/mL (Burnham et al. 2014). Although they are of great advantage, paper‐based sensors have limitations in terms of accuracy, resolution, sensitivity and multiplexed detection (Gosselin et al. 2017).

As advantageous with paper‐based devices, lateral flow assay devices are highly commercialized due to low cost, simplicity, specificity and portability. *P. aeruginosa* harmful genes were found in drinking water using a lateral flow test strip with numerous sensing zones (Chen et al. 2016). Due to their accessibility, low cost, high throughput, ease of use, low sample demand, speed, and on‐site applicability, microfluidic devices are also gaining popularity (Busa et al. 2016). Park et al. (Park and Yoon 2015) reported the use of microfluidic device on site for the detection of *E. coli* in aqueous samples. There has been reported advancement in detection of pathogens using microfluidic technique due to integration of microfluidic chips with other analytical techniques (Kumar et al. 2019).

Although, a great progress has been recorded in the use of nanosensors, some challenges still exist. The complexity of biological samples is a current challenge as there is a possibility of the presence of nontarget compounds in matrices which leads to inaccurate results such as underestimating or overestimating the concentration of the target analyte. This can be further complicated by sensor fouling, especially when the sample matrix includes proteins (Kurup and Ahmed 2023).

The development of highly specific, multi‐enzyme mimicking nanozymes could lead to the creation of highly sensitive and low‐biofouling sensors (Kurup and Ahmed 2023). Also, due to internal interactions and environmental influences, two or more enzyme‐like activities might appear under the same or comparable circumstances (Niu et al. 2022). Additionally, nanozymes for example are not uniform in size and composition and this can be a challenge in their use as sensing agent and to correct the effect of these, there is a need for advanced computation assisted technology (Singh 2019). If these currently existing challenges in the use of nanosensors can be addressed, they are promising point‐of‐use detection methods for screening the quality of water before consumption or household use. With better water quality monitoring, there will be reduced consumption of contaminated water, thereby reducing the outbreak of waterborne diseases and improving the health of individuals. The detection of microbial contaminants in water, if targeting the reduction of the health burden of waterborne pathogens, cannot rely on laboratory‐based analysis methods. Products are needed that can be used by individuals real time, thus putting their safety in their hands. Therefore, the use of nanosensors is a promising approach to the real‐time detection of water contamination and global health through safe water use.

## 7 Conclusion

The need for safe water cannot be overemphasized due to the implicated health burden of consuming and using contaminated water. There exist cultural and molecular methods that have been used for the detection of water contaminants. Although these methods have been successfully used in the detection of pathogens, sensors are better analytical devices; faster, sensitive, specific and easy to use. The use of nanosensors has given better advantage in detection of microbial contaminants and should also be improved for easier and faster detection and analysis. Multiplex analysis of pathogens and not just bacteria is necessary for the achievement of safe water for the use of the populace. Nanosensors should be targeted for more point‐of‐use purpose to make for instant assessment of water quality.

## Author Contributions

**Adeyemi O. Adeeyo:** conceptualization, writing – original draft, writing – review and editing. **Joshua N Edokpayi:** writing – review and editing, validation. **Mercy A. Alabi:** writing – original draft. **Joshua A. Oyetade:** writing – original draft. **Eunice Ubomba‐Jaswa:** validation. **Penny Jaca:** validation. **Rachel Makungo:** validation, writing – review and editing.

## Ethics Statement

The authors have nothing to report.

## Conflicts of Interest

None declared.

## Supporting information

Table S1: Different methods of detection of microbes using nanoparticles.

## Data Availability

Data sharing not applicable to this article as no datasets were generated or analyzed during the current study.
