# Supplementary material for: Detection of Microbial Contaminants in Water: Conventional Methods, Pragmatic Alternatives, and Nanosensing Techniques
Source: Microbiologyopen. 2025 Dec 8;14(6):e70057. doi: 10.1002/mbo3.70057 (PMC12685762; doi:10.1002/mbo3.70057)
Supplement: Supplementary file 1 — Table S1: Different methods of detection of microbes using nanoparticles. [file MBO3-14-e70057-s001.docx]

Appendix 1

Table S1: Different methods of detection of microbes using nanoparticles

| Nanoparticle based sensors | Nanomaterials | Microbe(s) detected | Detection method | References |
| --- | --- | --- | --- | --- |
| Carbon-based nanomaterial | GO-based Nps | *E. coli* K12  *Escherichia coli*  algal toxin  *C. parvum* oocyst wall  *Escherichia coli* | Graphene  Electrochemical  Electrochemical  Florescence  Electrochemical Impedance spectroscopy | (Jain et al., 2019; Karuppiah et al., 2021; So et al., 2008; Unni et al., 2017) |
|  | CNT-based | Bacteria  *E. coli* DH5a  *Escherichia coli*  MS2 *bacteriophage* | Field-effect transistors  Electronic  Electronic  Electrochemical | (Mao et al., 2014; Prieto-Simón et al., 2015; So et al., 2008; Yamada et al., 2014) |
| Metal and Metal oxide | Ag^+^  AgNps | *Salmonella Typhi*  *Salmonella Typhi*  *Salmonella typhimurium*  *Salmonella typhimurium* | Colorimetric  Electrochemical  Colorimetric  Colorimetric | (Kumar et al., 2020; Singh et al., 2019) |
|  | AuNps | Pesticides, bacteria, viruses, and protozoa  Colorimetric based  gG protein  Nucleic acids and influenza  *Listeria monocytogenes*  *E. coli*  *E. coli, V. cholerae, Pseudomonas aeruginosa, Xanthomonas campestris*  *P. aeruginosa*  *C. parvum* | Surface-enhanced Raman  Field-effect transistors  Bacterial cell DNA  Colorimetric  Colorimetric  Colorimetric  Colorimetric  Aptamer | (Huang et al., 2020; Iqbal et al., 2015; Jarvis and Goodacre, 2008; Peng and Chen, 2019; Reddy et al., 2022; Vilela et al., 2012) |
|  | Magnetic nanoparticle (MNps) | *Mycobacterium bovis,* *Influenza A*  *Salmonella enterica*  *Escherichia coli*  *S. aureus, Vibrio parahaemolyticus* | Magnetoresistance  Hydrodynamic property changes  Electrochemical  Colorimetric | (Fu et al., 2020; Guo et al., 2021; Jiang et al., 2016; Liu et al., 2020) |
|  | Zinc oxide nanoparticles | *Escherichia coli O157* | conductometric | (Mutlaq et al., 2021) |
| Silica nanoparticles (SiNps) | Silicon nanowires (SiNWs) | *Influenza* and Nucleic acids  *Escherichia coli*  *Entamoeba histolytica*  *Staphylococcus aureus* | Chemiresistors  Electrochemical  Fluorescent  dielectrophoresis and aptamer-fluorescent | (Hemadi et al., 2015; Mathelié-Guinlet et al., 2019; Shangguan et al., 2015; Wang et al., 2018; Zhu et al., 2018) |

Key: Nps- Nanoparticles, MNps-Magnentic nanoparticles, NWS- Nanowires
